# No Benefit of a Pediatric Screening in Discovering Reduced Visual Acuity in Children: Experiences from a Cross-Sectional Study in Germany

**DOI:** 10.3390/ijerph17103419

**Published:** 2020-05-14

**Authors:** Heike M. Elflein, Roman Pokora, Denis F. Müller, Klaus Jahn, Katharina A. Ponto, Susanne Pitz, Norbert Pfeiffer, Alexander K. Schuster, Michael S. Urschitz

**Affiliations:** 1 Department of Ophthalmology, Universitätsmedizin der Johannes Gutenberg-Universität Mainz, 55131 Mainz, Germany; denisfriedrichmueller@gmail.com (D.F.M.); katharina.ponto@unimedizin-mainz.de (K.A.P.); norbert.pfeiffer@unimedizin-mainz.de (N.P.); alexander.schuster@unimedizin-mainz.de (A.K.S.); 2 Division of Paediatric Epidemiology, Institute of Medical Biostatistics, Epidemiology and Informatics, Universitätsmedizin der Johannes Gutenberg-Universität Mainz, 55131 Mainz, Germany; pokora@uni-mainz.de (R.P.); urschitz@uni-mainz.de (M.S.U.); 3 Ministry for Social Affairs, Labour, Health and Demography of Rhineland-Palatinate, Department of Public Health Services, Hygiene and Infection Control, 55131 Mainz, Germany; Klaus.Jahn@msagd.rlp.de; 4 Department of Ophthalmology, Bürgerhospital und Clementine Kinderhospital, 60318 Frankfurt/Main, Germany; s.pitz@buergerhospital-ffm.de

**Keywords:** amblyopia, preschool health examination, pediatric eye screening

## Abstract

Background: The newly introduced German pediatric screening examination at the end of the third year of life (U7a) incorporates visual function testing in particular; there is no ophthalmic screening during childhood in Germany. The purpose of this study is to investigate the relationship between participation in U7a and visual function at the preschool health examination (PHE) in the sixth year of life. Methods: This study evaluated PHE data from school enrollment years 2009/2010 to 2014/2015 of Rhineland-Palatinate, Germany. Visual acuity (VA) at PHE was assessed with Rodenstock visual acuity test device (tumbling E) wearing glasses if present. The relationship between participation in U7a and VA <0.7 at PHE was calculated for reduced monocular and binocular VA using multiple logistic regression adjusted for potential confounders. Results: Data from 189,704 children (91,041 girls) in 35 out of 36 districts were included. The first children to participate in U7a were enrolled in 2011/2012 school year. In total, 90,339 children (47.6%) had U7a before PHE, while 99,365 (52.4%) had not. VA <0.7 in at least one eye was measured at PHE in 8429 (4.4%) children, and in both eyes in 4345 (2.3%) children. Participation in U7a was not associated with VA <0.7 at PHE (odds ratio 0.99; 95% confidence interval: 0.94–1.04). Conclusions: The proportion of children with VA <0.7 at PHE was high. No beneficial effect of newly introduced German U7a pediatric screening examination was found for reduced VA at PHE.

## 1. Introduction

The aim of a preventive eye examination in childhood, regardless of the professional group carrying out the examinations, is early detection of visual disorders, which often require prompt treatment. One focus of eye examinations in childhood should be to detect amblyopia or ‘lazy eye’. Amblyopia is the most common cause for reduced visual acuity in childhood [1,2] adolescents, and young adulthood and it usually affects one eye. It occurs in early childhood due to insufficient development of the visual system without any apparent structural abnormality or organic problems of the eye. Around half of the amblyopic cases are caused by anisometropia (i.e., a different refractive error in both eyes that is not noticeable in everyday life). If there is no therapy (prescription of suitable glasses, occlusion therapy if necessary), the higher ametropic eye usually does not learn to see clearly during early childhood development of the visual cortex. This development of the visual cortex cannot be adequately achieved in adulthood and, as consequence, therapy in adulthood is not promising. If treated too late or left untreated, amblyopia may remain a problem throughout life. In addition to reduced monocular vision, individuals with amblyopia suffer from subnormal or non-binocular perceptual performance [3] and also from sensory deficits such as reduced position acuity and contrast sensitivity [4]. It might also affect specialized skills such as acquiring a commercial driver’s license. The population prevalence of amblyopia in Germany is estimated to be 5.6% at the age of 35–44 years [5]. This prevalence is high by European standards: in Sweden [6] and Denmark [7], it was estimated to be only 1.1% and 1.4%, respectively. In these two countries, preventive eye examinations were established at preschool age more than 40 years ago. In Denmark the prevalence of amblyopia decreased by four-fold.

In addition to amblyopia, a preventive pediatric eye examination should also detect rare eye diseases, such as childhood cataract (population prevalence 0.03% [8]) or congenital glaucoma (prevalence even lower), all of which should be diagnosed and treated as early as possible to ensure optimal visual development [9,10].

In Germany, 10 mandatory pediatric preventive health examinations are provided from birth up to the beginning of the sixth year of life aiming for the early detection of any diseases or developmental problems. They are called U1 to U9 and take place at a pediatrician chosen by the parents. U is for the German word “Untersuchung” (examination), and the contents of each examination are clearly defined. For further details, see Table 1. All health examinations include at least an inspection of the eyes. The U7a (at the end of the child’s third year of life), U8 (at the end of the child’s fourth year of life), and U9 (at the beginning of the child’s sixth year of life) are further intended to discover vision disorders at preschool age. Thus, a vision test should be at least part of these pediatric health examinations. However, an ophthalmological vision screening with a detailed examination of the eyes and visual functions for children is currently not an obligatory part of the covered services of the statutory health insurance in Germany. One reason for this fact might be that in 2007, the German Institute for Quality and Efficiency in Health Care (German: “Institut für Qualität und Wirtschaftlichkeit im Gesundheitswesen” (IQWIG)) found out that there was currently no evidence or indication of benefits of an ophthalmological screening at preschool age [11]. This statement was confirmed by the IQWIG in a rapid report in 2015 after renewed examination [12]. In particular, IQWIG claimed, that there is not only a small number of studies with poor quality and conflicting results, but also a lack of research into potentially harmful aspects of vision screening. Nonetheless, in 2008, the German Federal Joint Committee (German: “Gemeinsamer Bundesausschuss” (GBA); the highest decision-making body of the joint self-government of physicians, dentists, hospitals, and health insurance funds in Germany) added the U7a at the end of the child’s third year of life to the pediatric preventive health examinations. It includes, in particular, testing of visual acuity. In Rhineland-Palatinate, as in other German federal states, there is a binding invitation and registration system for these preventive health examinations. In addition to these examinations, an obligatory preschool health examination (PHE) performed by public health physicians is carried out in Germany during the sixth year of life to detect and resolve school-relevant health impairments before entering school; it includes testing of monocular visual acuity as well. However, as a preventive examination, its task is not to find out the reason for a possibly reduced visual acuity (which most commonly is amblyopia [1,2]).

Almost no study has dealt with the effectiveness of preventive eye examinations in childhood (i.e., whether children with visual impairments are effectively detected in real-life). Jonas et al. concluded in a review of 40 studies that there are no studies directly testing the effectiveness of eye screening programs [13]. The present study aims to explore whether participation in the newly introduced U7a is effective and results in a lower proportion of children with reduced visual acuity at the time of the PHE.

## 2. Methods

### Setting, Participants, and Study Design

This study used data from preschool children in the German Federal State of Rhineland-Palatinate, an area of approximately 19,854.21 km^2^ with 4 million inhabitants in 2018. This cross-sectional study comprised all children registered for school entry within a 6-year period, from 2010 to 2015. Mandatory PHE took place in 24 administrative districts and 12 independent cities of Rhineland-Palatinate. It was carried out by public youth health physicians employed by the respective regional Departments of Public Health before school enrollment; standardized operating procedures were followed, and staff trained in a regular manner. With the exception of one district (Altenkirchen), all information was uniformly documented, and data were provided in anonymous form by the Federal Ministry for Social Affairs, Labour, Health, and Demography of Rhineland-Palatinate.

#### 2.1.1. Contents of the PHE

Children and their parents attended a compulsory medical examination and a hearing and vision screening at the PHE. In addition, a survey of demographic and health factors via a parental questionnaire took place. The parental questionnaire included—among other topics—information on the highest school-leaving qualifications of both parents, languages spoken at home, any need to wear glasses, and visits to an ophthalmologist in the past 12 months. The preventive pediatric health examination booklet was provided by parents and used to document the uptake of the preventive health examinations ‘U1′ to ‘U9′.

The PHE vision screening test was performed monocularly (separately for each eye) with glasses on a Rodenstock vision test device (R11 or R21; visual sign: test disc 120: tumbling E) for distance and proximity. Visual acuity was recorded in decimal form and predefined categorized as “<1.0 but >0.7” and “<0.7” and conspicuous findings related to spatial and color vision were documented.

#### 2.1.2. Contents of U7a

The main contents are tests of visual acuity, monocular (separately for each eye) and of stereoacuity. In addition to an inspection of the eyes to rule out morphological abnormalities, nystagmus or any head posture, U7a also includes an evaluation of the pupil (comparison of size, shape, light reaction OD/OS).

#### 2.1.3. Statistical Analyses

All children who underwent an eye test at the PHE were included in this explorative analysis. Absolute and relative frequencies were stratified for visual acuities <0.7 (unilaterally or bilaterally; each with a recommendation for an ophthalmological examination) for the entire sample, for each enrollment year, and for participation in the preventive health examination known as the U7a.

Odds ratios (ORs) and their 95% confidence intervals (95% CI) were calculated using multiple logistic regression analysis to examine the relationship between participation in the U7a examination and the presence of unilateral or bilateral visual acuity <0.7 at the PHE. Potential confounders were previously determined using a causal diagram (“Directed Acyclic Graph”). ORs were adjusted for sex, age (in months), highest parental education, household language, and wearing glasses in the last 12 months. *p*-Values are reported in the context of this explorative analysis but are only for descriptive purpose. Thus, a significance level was not defined and adjustments for multiple testing not performed. All analyses were carried out using the software package R (version 0.99.896).

## 3. Results

Of 204,123 children examined, 160,122 children (78.4%) were included in the analysis with data on U7a and visual acuity (Table 1; demographic characteristics). For 13,152 (6.4%) children, there was no precautionary book available, for 1267 (0.6%) children the examination was incorrectly coded, and for 5719 (2.8 %) children, there was a lack of U7a data. Furthermore, 23,863 (11.7%) additional children had missing data on visual acuity. Among the included children, there were 76,721 girls, 10,760 (6.7%) children wore glasses, and 41,394 children (25.9%) had been to an ophthalmologist at least once before the PHE. For 144,585 (90.3%) children, the language spoken at home was German.

Visual acuity <0.7 in at least one eye was found in 8185 children (5.1%). This proportion increased over time (Table 1). In 4236 (2.7%) children, visual acuity was low in both eyes; in 3949 (2.5%) children it was low in only one eye. The proportion of children who had taken part in all recommended preventive health examinations including the U7a increased during the observation period (Table 1).

The first children who participated in the U7a started school in 2011/12. U7a was performed in 78,491 children (49.0%) before the PHE, and it was not performed in 81,631 (51.0%) children. The U7a had not yet been introduced for 56,141 (35.1%) children and thus these children were included in the no U7a study group. In the course of the observation period, the U7a participation rate increased from 38.5% in 2011/12 to 91.7% in 2014/15.

**Figure 1:**
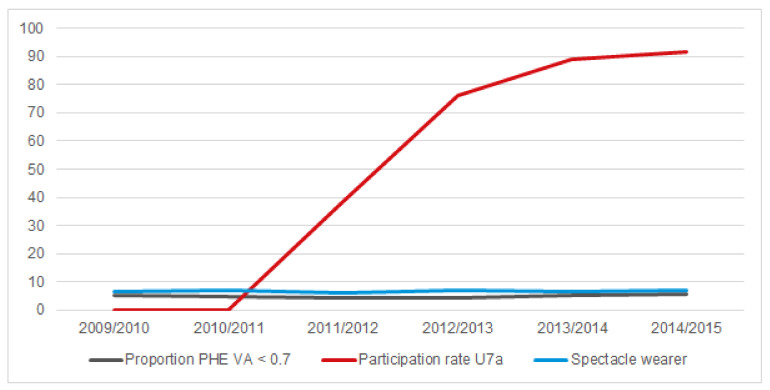
Time trends for the proportion of children with mon- or binocular visual acuity <0.7, the U7a participation rate, and the proportion of spectacle wearers for each year, all %. PHE preschool health examination in the sixth year of life; VA visual acuity; U7a newly introduced paediatric screening examination at end of third year of life.

Before the PHE, 25.5% of U7a participants and 26.2% of U7a non-participants had been to the ophthalmologist. Among the U7a participants, the proportion of children wearing spectacles was 6.8% and among the U7a non-participants it was 6.6%; see Figure 1).

### 3.1. Unilateral Low Visual Acuity

**Figure 2:**
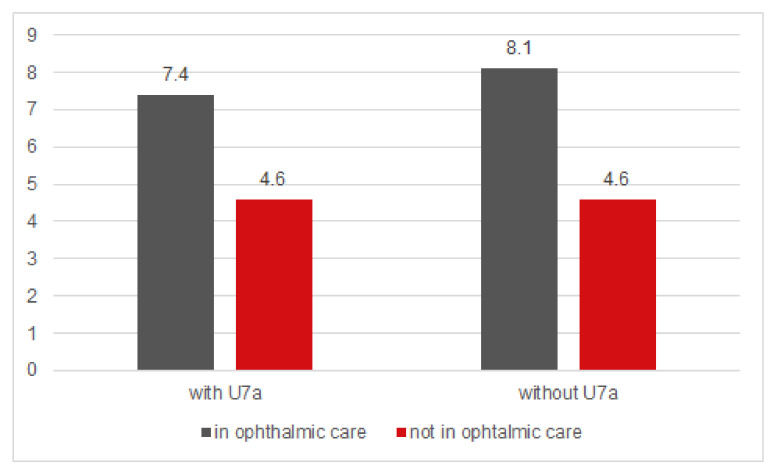
Prevalence (%) of children with PHE visual acuity <0.7 (mon- or binocular) stratified by U7a participation. PHE preschool health examination; VA visual acuity; U7a newly introduced paediatric health examination at end of third year of life.

In U7a participants, 1943 (2.2%) children had unilateral visual acuity <0.7. Of these children, 699 (36.0% of the 1943 U7a participants with reduced visual acuity) had been to an ophthalmologist before the PHE. In U7a non-participants, the proportion of children with reduced visual acuity on one eye was 2.14% (N = 2006); of these, 723 (36.0% of the 2006 U7a non-participants with poor visual acuity) reported a prior visit to an ophthalmologist (Figure 2).

In adjusted regression analysis there was no association between participation in the U7a and reduced unilateral visual acuity (<0.7) at the PHE (adjusted OR: 1.02; 95% CI: 0.95–1.10; *p* = 0.60; N = 117.591).

### 3.2. Low Visual Acuity in Both Eyes

In 2.2% (N = 1997) of the U7a participants, visual acuity <0.7 was measured in both eyes. In 35.7% of these children (N = 712), a visit to an ophthalmologist had been made before the PHE. Reduced visual acuity in both eyes was found in 2.4% (N = 2239) of the children without U7a participation; in 37.3% of them a visit to an ophthalmologist was documented. In adjusted regression analysis there was no association between participation in the U7a and bilateral reduced visual acuity (<0.7) at the PHE (adjusted OR: 0.96; 95% CI: 0.89–1.03; *p* = 0.26; N = 117,748).

## 4. Discussion

In our study, participation in the U7a pediatric preventive health examination at the end of the third year of life was not associated with a lower proportion of children with reduced visual acuity at the PHE in the sixth year of life (Table 2). There was also no increase in having consulted an ophthalmologist or in wearing spectacles in U7a participants compared to U7a non-participants. We, hence, could not find any beneficial effects of the U7a examination on the ophthalmologic care or visual acuity at the time of the PHE. However, the mandatory SEU program requires a cut-off at a visual acuity of 0.7; we cannot rule out that U7a might be more effective for more severe visual impairment.

Amblyopia is the leading cause for reduced visual acuity in childhood [1,2], however neither PHE nor U7a are intended to find out the reason for any reduced visual acuity. Observational studies such as ours are subject to several sources of bias, in particular due to the lack of suitable control groups or due to residual confounding (i.e., neglecting important predictor variables). The methodological strength of the present study lies in the fact that it is a so-called “natural experiment” and thus a quasi-experimental study, because the intervention was introduced from “outside” in a controlled manner and allowed repetitive observations before and after the introduction of the U7a. As a result, the intervention group is more structurally similar to the control group and the observed associations less susceptible to bias and confounding. The study design allowed for an investigation of the relationships between U7a participation and the outcomes of interest (i.e., ophthalmologist visit, eyeglass care, vision disorder at the PHE) through repetitive cross-sectional data over several years before and after the intervention’s introduction. In addition, the effect estimates were adjusted for important confounders, so that the observed zero effect of the U7a is probably largely unbiased. However, U7a, like all other pediatric screening examinations, takes place with a pediatrician chosen by the parents; even though the requirements and contents of these examinations are clearly defined, there is no specific nor mandatory training.

Within the scope of the eye examination part of the U7a, visual dysfunctions and conspicuous eye findings requiring an ophthalmological examination or potential treatment were expected to be discovered. A higher number of ophthalmological referrals or spectacle wearers could be regarded as an indirect indication of a benefit of the U7a. Nevertheless, the proportion of spectacle wearers among the U7a participants was just as high as among the U7a non-participants. The same applies for ophthalmologist visits prior to the PHE. In comparison, a study of kindergarten children in Germany from 1998 showed a similar proportion of spectacle wearers [14].

The overall proportion of children with reduced visual acuity before starting school was 5.1%. During the 2009/2010 to 2014/2015 school enrollment years, this proportion was highest at 5.9% in the last (i.e., 2014/2015) school enrollment year. At the same time, the participation rates in preventive health examinations rose, so this cannot explain the change in prevalence (Table 1, Figure 2).

One reason for the apparent lack of effectiveness of the U7a with regard to vision could be poor cooperation of children due to their young age. In about half of the children with reduced visual acuity, visual acuity <0.7 was measured in only one eye. Children with reduced visual acuity on one side are typically inconspicuous in everyday life because the poor visual acuity on one side can be compensated with the other side. This underlines the importance of a correctly performed visual acuity test: visual acuity must be tested monocularly. If this is not successful, the eye test must be classified as conspicuous and at least repeated within a short time. For example, Brückner’s test (red fundus reflexes in the pupil with a direct ophthalmoscope) is helpful for all pediatric screening examinations; with this simple examination procedure, media opacities, strabismus, and different refractive errors can be detected.

Due to the different methods and ages of the children examined, a direct comparison with studies from other European countries is difficult. However, the population prevalence of preschool children, who see poorly in our study appeared to be high. In the Netherlands, a cohort study of 7-year-old children found only 1.6% with visual acuity ≥0.2 logMAR (comparable to ≤0.63 Snellen Visus). One obvious explanation is that five eye examinations are carried out on Dutch children in the first two years of life. Better compliance may also reduce the proportion of children with poor eyesight due to the slightly older age of the Dutch children, who were examined for visual acuity [15,16].

A British study tested the monocular visual acuity of 7.5-year-old children; after vision screening at the age of 4–5 years, the proportion of children with visual acuity <6/9 (<0.67) was 1.9%. If this screening was not performed, the proportion was 3.4% [17]. For the visual acuity criterion 0.5 (0.3 logMAR), the proportions were 0.7% and 1.3% with and without screening, respectively. Extensive orthoptic screening in the first 37 months of life in Great Britain showed a clear effect on amblyopia prevalence at the age of 7.5 years. After orthoptic screening at the ages of 8, 12, 18, 25, 31, and 37 months, this was 0.6%. If only one orthoptic examination was performed at the age of 37 months, the prevalence of amblyopia was 1.8%. One of the criteria for amblyopia is visual acuity <0.5 (greater than 0.3 logMAR) in the better eye, making a comparison with the present study difficult.

In summary, these European data point to a positive effect of an appropriate early vision screening and thus contradict the present study. However, screening examinations and diagnostic criteria vary greatly from country to country as does the profession of the respective examiner. A “gold standard” for an eye screening in childhood is missing as well as valid studies to verify the effectiveness of a particular diagnostic test.

There are several possible causes for the high proportion of children (2.7%) with bilateral visual acuity <0.7 at the PHE. A bilateral reduction in visual acuity may be due to higher symmetrical ametropias, the prevalence of which is around 30% in children of this age [18,19,20]. Eye pathologies relevant to visual acuity, such as bilateral childhood cataract or early retinal dystrophy, have low prevalence rates; for cataracts it is about 0.03% [8]. The children’s lack of concentration and cooperation may be another important reason leading to false positive screening tests.

## 5. Conclusions

The proportion of children with visual acuity <0.7 at the PHE was high compared to other European countries. A beneficial effect of the newly introduced German pediatric screening examination known as the U7a was not found for reduced visual acuity at the PHE. However, the Federal Joint Committee has updated and improved the “Directive on the early detection of childhood diseases” (German: “Kinder-Richtlinie”) in the meantime. Pediatricians were sensitized to the importance of the eye examination within the U7a and other preventive health examinations. It remains to be seen whether this can reduce the proportion of children with reduced visual acuity at preschool age in the future.

## Figures and Tables

**Table 1:** Demographic information.

Year	2009/2010	2010/2011	2011/2012	2012/2013	2013/2014	2014/2015	Total
N	28,454	27,687	22,403	26,955	27,587	27,036	160,122
Female (%)	13,584 (47.8)	13,165 (47.5)	10,859 (48.5)	12,855 (47.7)	13,256 (48.1)	13,002 (48.1)	76,721 (47.9)
Average age (years) at PHE	5.8 ± 0.4	5.8 ± 0.4	5.8 ± 0.3	5.8 ± 0.4	5.8 ± 0.4	5.8 ± 0.4	5.8 ± 0.4
Proportion (%) PHE VA <0, 7 (monocular)	679 (2.39)	653 (2.35)	511 (2.28)	602 (2.23)	708 (2.56)	796 (2.94)	3949 (2.46)
Proportion (%) PHE VA <0, 7 (binocular)	794 (2.79)	713 (2.57)	507 (2.26)	634 (2.35)	778 (2.82)	810 (2.99)	4236 (2.65)
U7a participation rate (%)	-	-	38.46	75.97	89.18	91.69	49.02
Proportion (%) of children participating in all screening examinations (U1–U9 without U7a)	74.04	77.98	82.59	85.56	87.41	89.72	82.8

PHE: preschool health examination in the sixth year of life; VA: visual acuity; U7a: newly introduced pediatric screening examination at end of third year of life.

**Table 2:** Proportion of children with reduced VA at PHE.

Total Number of Participants (N)	160,122
**Number of participants with U7a (N)**	78,491
proportion (%) of children with PHE VA <0.7 (mon- or binocular)	5.02
proportion (%) of children with PHE VA <0.7 (monocular)	2.48
proportion (%) of children with PHE VA <0.7 (binocular)	2.54
**Number of Participants without U7a (N)**	81,631
proportion (%) of children with PHE VA <0.7 (mon- or binocular)	5.20
proportion (%) of children with PHE VA <0.7 (monocular)	2.46
proportion (%) of children with PHE VA <0.7 (binocular)	2.74

PHE: preschool health examination; VA: visual acuity; U7a: newly introduced pediatric health examination at end of third year of life.

## Abbreviations

VA	visual acuity
PHE	preschool health examination
U7a	preventive health examination at the end of the third year of life
U8	preventive health examination at the end of the fourth year of life
U9	preventive health examination at the beginning of the sixth year of life
IQWIG	German Institute for Quality and Efficiency in Health Care (“Institut für Qualität und Wirtschaftlichkeit im Gesundheitswesen”)
CI	confidence intervals
